# Spectral-domain OCT measurements in obesity: A systematic review and meta-analysis

**DOI:** 10.1371/journal.pone.0267495

**Published:** 2022-04-27

**Authors:** Mohammad Amin Salehi, Amirali Karimi, Soheil Mohammadi, J. Fernando Arevalo

**Affiliations:** 1 School of Medicine, Tehran University of Medical Sciences, Tehran, Iran; 2 Wilmer Eye Institute, Johns Hopkins University School of Medicine, Baltimore, United States of America; Icahn School of Medicine at Mount Sinai, UNITED STATES

## Abstract

**Background:**

Previous studies proposed possible applications of spectral-domain optical coherence tomography (SD-OCT) measurements in prognosticating pathologies observed in overweight/obesity, including ocular, vascular, and neurologic consequences. Therefore, we conducted a systematic review and meta-analysis to investigate the changes in the in SD-OCT measurements of the patients with higher body mass index (BMI) compared to normal weight individuals.

**Materials and methods:**

We conducted a systematic search on PubMed, Scopus, and Embase. The search results underwent two-phase title/abstract and full-text screenings. We then analyzed SD-OCT measurements differences in patients with high BMI and controls, and performed meta-regression, sub-group analysis, quality assessment, and publication bias assessment. The measurements included macular thickness, cup to disc ratio, ganglion cell-inner plexiform layer (GC-IPL) and its sub-sectors, RNFL and peripapillary RNFL (pRNFL) and their sub-layers, and choroidal thickness and its sub-sectors.

**Results:**

19 studies were included in this meta-analysis accounting for 1813 individuals, 989 cases and 824 controls. There was an overall trend towards decreased thickness in high BMI patients, but only two measurements reached statistical significance: temporal retinal nerve fiber layer (RNFL) (Standardized mean difference (SMD): -0.33, 95% confidence interval (CI): -0.53 to -0.14, p<0.01) and the choroidal region 1.0 mm nasal to fovea (SMD: -0.38, 95% CI: -0.60 to -0.16, p<0.01).

**Conclusion:**

Some ocular layers are thinner in patients with higher BMI than the controls. These SD-OCT measurements might correlate with adverse events related to increased body weight and have prognostic abilities. As SD-OCT is a robust, rapid and non-invasive tool, future guidelines and studies are needed to evaluate the possibility of their integration into care of the patients with obesity.

## 1. Introduction

Obesity is among the most important global health challenges worldwide [1]. The global burden of obesity is growing with its increasing prevalence, as the number of patients with obesity almost tripled from 1975 to 2016 [2]. In 2016, 603.7 million adults and 107.7 million children had obesity, and more than 4.0 million deaths were attributed to high body mass index (BMI) [3]. This condition serves as a risk factor for wide range of diseases, including cardiovascular diseases [4], several cancers [5], chronic kidney disease [4], diabetes mellitus [6], hypertension [6], and various musculoskeletal disorders [7]. Cardiovascular diseases are responsible for more than two thirds of the high BMI-related deaths [3]. Therefore, it is crucial to find non-expensive, non-invasive, and technically simple screening factors that identify and prognosticate the complications of obesity, especially cardiovascular consequences, and has been a challenge for many years [8]. Obesity also put the patients at higher risk for many ocular complications, including diabetic retinopathy, glaucoma, age-related cataract, age-related maculopathy, and involvements of various ocular layers [9–14], requiring specific attentions to predict, diagnose, and manage these adverse outcomes.

Development of spectral-domain optical coherence tomography (SD-OCT) provided an opportunity to assess the ocular layers with high precision and reproducibility [15]. High-resolution SD-OCT can obtain three-dimensional images to analyze the volume and thickness of all the retinal and choroidal layers [16]. SD-OCT outperforms the first-generation time-domain OCT (TD-OCT) in several aspects, including greater accuracy and details, higher sensitivity, improved scan resolution, superior signal-to-noise ratio, and faster speed [15, 17].

Recent studies introduced SD-OCT measurements of retina and choroidal thickness as predictors of higher risk of concurrent or future vascular pathologies in patients with obesity [18–20]. As mentioned above, SD-OCT is a robust and non-invasive imaging method to detect the slightest changes in these ocular layers [15, 16]. In light of the possible opportunities offered by SD-OCT measurements in obesity, we conducted a systematic review and meta-analysis to investigate the changes in the retinal and choroidal SD-OCT measurements in patients with higher BMI compared to healthy normal weight individuals.

## 2. Materials and methods

### 2.1. Study design

We conducted a systematic review and meta-analysis to compare SD-OCT measurements in individuals with overweight/obesity and healthy normal weight controls according to the Preferred Reporting Items for Systematic Reviews and Meta-Analyses (PRISMA) checklist [21]. TD-OCT methods studies were not included in this meta-analysis due to its several differences and disadvantages compared to SD-OCT. Two authors (MA.S.) and (A.K.) independently performed a two-phase screening process to select the eligible articles based on the inclusion criteria. First, records identified through database search were screened based on the cohesion of titles and abstracts to inclusion criteria. The ineligible records in this step were excluded and the eligible ones entered another phase where we examined their full-texts to select the eligible studies. Discrepancies were solved through discussion with a third author (S.M.). This study was registered in the International Prospective Register of Systematic Reviews (PROSPERO) with the code of CRD42021238501.

### 2.2. Eligibility criteria

We included studies with the following features that were related to the aim of this article:

1. Original observational studies on human population, including case-controls, cohorts, and cross-sectional studies
2. Studies possessing at least two groups; a group of individuals with high BMI, and a control group comprised of healthy normal weight individuals to compare the findings with the cases.
3. Overweight and obesity established through BMI criteria (BMI ≥ 25)
4. Articles reporting the values for SD-OCT measurements as mean and standard deviation (SD), or values that could be converted to mean (SD) using previously established formulas, e.g. median and interquartile range or median and range.

Therefore, studies not related to our aim, abstracts and conference abstracts, case reports and case series, review articles, pure animal or laboratory studies, and those lacking control groups were considered ineligible and were excluded.

### 2.3. Search term

We conducted a title/abstract search using online databases of PubMed, Scopus, and Embase on 21^st^ February, 2021, using the following keywords:

- [Optical Coherence Tomography] OR [OCT Tomography] OR [Tomography, Optical Coherence] OR [Tomography, OCT] OR [Coherence Tomography, Optical]
- [Obese] OR [Overweight] OR [Obesity] OR [Body Mass Index] OR [Index, Body Mass] OR [Quetelet Index] OR [Index, Quetelet] OR [Quetelet’s Index] OR [BMI]
- [A] AND [B]

### 2.4. Data extraction

Two researchers (MA.S.) and (A.K.) independently extracted all the necessary data of the eligible studies in an excel sheet, including the country, design, inclusion/exclusion criteria, OCT model, age/sex matching status, definition and number of cases and controls, male percentage, mean age, factors related to weight, metabolic profile and insulin resistance, intraocular pressure, mean axial length, mean spherical equivalent, and all the SD-OCT measurements. A third researcher (S.M.) reviewed the extracted data and checked for any problems in the process.

We contacted the authors if further clarifications were required regarding their data or full-texts of their studies were unavailable.

### 2.5. Data analysis

Mean (SD) was used to calculate the standardized mean difference (SMD) and its confidence interval (CI) for all the variables. We converted possible reported median values to mean (SD) using the formula previously described by Wan et al. [22], and included the studies in meta-analysis. However, we also performed sensitivity analysis omitting the studies that reported median instead of mean, only including those in the sensitivity analysis that originally reported mean (SD) values.

We used Higgins I^2^ test to assess the heterogeneity of the studies reporting the SD-OCT measurements. Variables with I^2^ values above 40% was analyzed using random-effect models, while the others underwent fixed-effect analysis.

Egger’s test and funnel plots helped us assess the potential publication bias for each of the measurements. We used trim and fill strategy to overcome the potential publication bias in the variables with asymmetric funnel plots that reached significant Egger’s test results.

We performed a meta-regression analysis on several variables to detect the potential confounders that affect the results, including study methods, participant baseline characteristics, some ocular parameters, and several variables related to metabolic syndrome and obesity. We also performed sub-group analyses based on the patient’s age group (children or adult), method of eye selection (single eye, both eyes, or mixed eyes), matching status of the studies, and SD-OCT model. Childhood was defined as younger than 18 years old, and adulthood as older than this age. An earlier study on SD-OCT measurements in Alzheimer’s disease found differences between various SD-OCT devices (e.g. Zeiss Cirrus HD, Heidelberg Spectralis, etc.) and changes in thicknesses of some layers between cases and controls [17]. This study encouraged us to provide these analyses taking into account the type of SD-OCT device utilized. Meta-regression and sub-group analyses were only performed on the variables with sufficient number of studies.

We chose p-value = 0.05 as the threshold for significance, and utilized Stata (StataCorp, Texas, version 16.0) for all the analyses in this study.

### 2.6. Quality assessment

We assessed the quality of the included studies using the Newcastle-Ottawa scale (NOS) tool [23], consisting of three major categories of selection, comparability, and exposure.

## 3. Results

**Fig 1:**
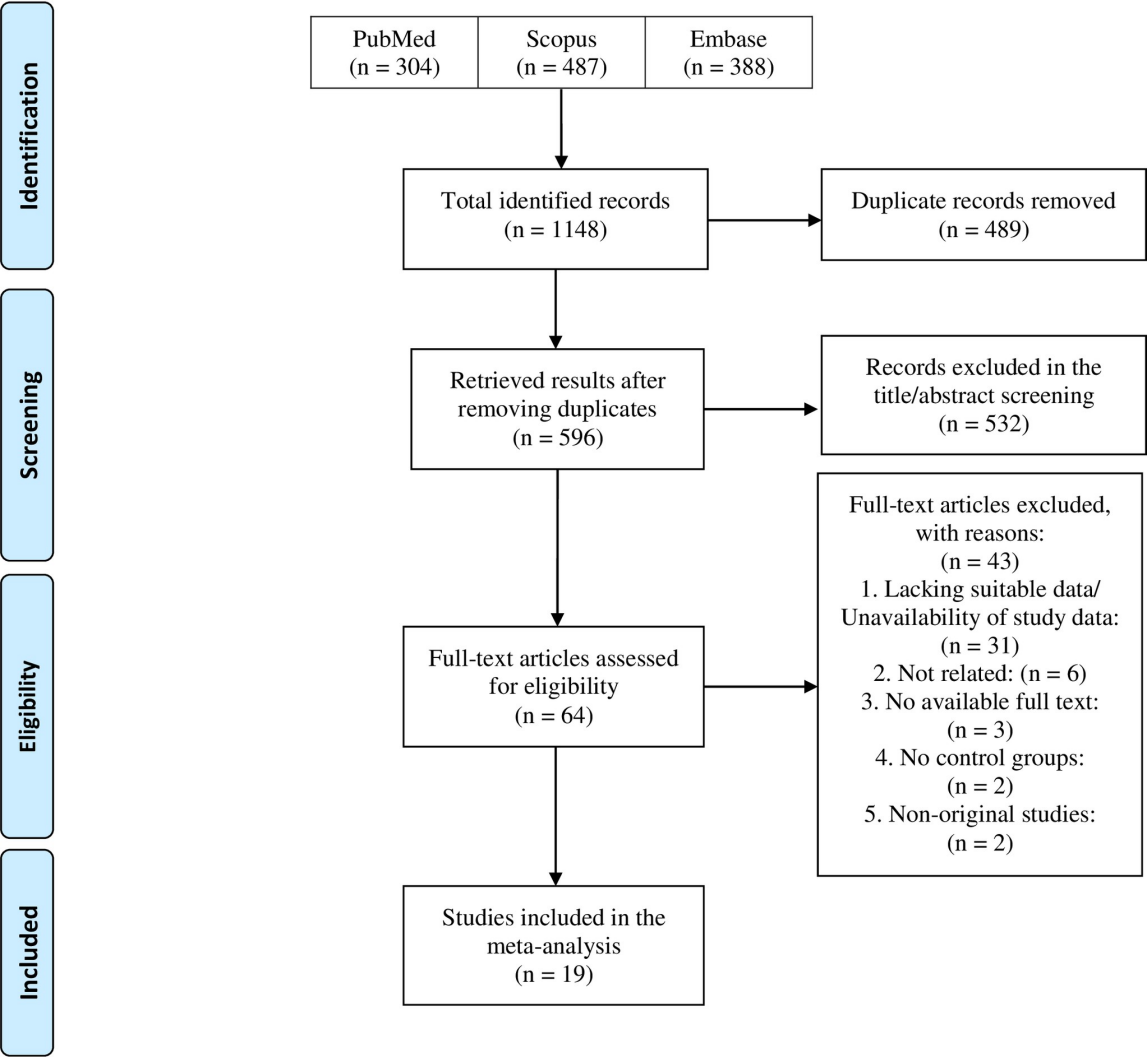
PRISMA flow diagram of the study’s selection process.

Our systematic search yielded 1148 records, of which 489 were duplicates. The remaining 596 studies underwent title/abstract screening and 532 studies were excluded based on their title/abstract. We screened the full texts of the remaining articles and 20 studies were initially eligible for the quantitative synthesis. Lee et al. [24] measured macula pigment optical density and central subfield thickness, but the number of studies were insufficient for conducting a meta-analysis on these parameters. Therefore, the remaining 19 studies were finally included for the meta-analysis. **Fig 1** illustrates the selection process in details.

We sent 31 electronic communications (e-mails) to the authors for several reasons, including no available full-texts, not mentioning mean or SD, or not mentioning the correlation between BMI of cases and controls and SD-OCT measurements. Five of the corresponding authors replied to our emails, 4 of them about association between BMI and OCT measurements and 1 on the unavailability of full-text. Finally, only 1 responded positively, (Uslu Dugan et al.) and provided us with the full-text of their study [25].

Of the 19 included studies in this meta-analysis, 5 measured macular thickness [9, 20, 26–28], 3 measured GC-IPL layer thickness [10, 29, 30], 5 measured RNFL thickness [12, 27, 28, 31, 32], and 7 studies measured peripapillary RNFL thickness [10, 11, 13, 25, 26, 29, 30], 5 measured cup to disc ratio [26–28, 30, 31], and 12 measured choroidal thickness [9, 14, 20, 25–28, 32–36]. All the studies reported their data as mean (SD), except one that reported some of the measurements in median (min-max) [26]. The total number of participants reached 1813, with 989 being in the overweight/obesity groups and 824 in the controls. The controls in all the studies were picked from healthy volunteers with normal BMI values (between 18.5 and 25). Some of the studies matched the participants for age and/or sex, depicted in **Table 1**. All the studies were cross-sectional or case-control. Nine studies matched the obesity and control groups in terms of age [9, 11, 27–30, 32, 34, 35], and 8 others matched their sex [9, 27–30, 32, 34, 35]. Nine studies analyzed a single eye of the subjects [9, 14, 20, 28–30, 32–34], 6 investigated both eyes [10, 12, 13, 25, 26, 35], 2 studies used a mixed approach (both single eyes and both eyes) [11, 31], and 2 others did not state it clearly [27, 36]. Nine utilized Zeiss Cirrus HD [9, 10, 13, 26–31], 8 studies used Heidelberg spectralis [11, 12, 20, 32–36], and 2 other studies benefited from other OCT models, including Topcon 3D-OCT [25] and Nidek RS-3000 [14], each in one study. 16 studies were from Turkey, and the remaining 3 originated from Portugal, Spain, and Thailand. **Table 1** depicts the characteristics of all the included studies.

**Table 1:** Characteristics, population, and discussed variable of the included studies.

Studies	Country	Number of Participants	Method of eye Selection	Mean Age	Male Percentage	Matched for:	SD-OCT Model	Macular thickness	Macular GC-IPL thickness	RNFL thickness	pRNFL thickness	Cup to disc ratio	Choroidal thickness
Öncül (2021)	Turkey	40 class I obese	Single	36	0	-	Heidelberg Spectralis	-	-	-	-	-	+
		44 controls		37.5	0								
Uslu Dogan (2020)	Turkey	50 obese	Both	24.6	100	-	3D OCT-2000 FA plus, version 8.20, Topcon, Tokyo, Japan	-	-	-	+	-	+
		50 controls		24	100								
Pekel (2020)	Turkey	41 obese	Single	11.5	29.3	Age/sex	Zeiss Cirrus HD	-	+	-	+	+	-
		41 controls		11.9	29.3								
Teberik (2019)	Turkey	101 morbid obese	Single	35.9	32.7	Age/sex	Heidelberg Spectralis	-	-	-	-	+	+
		95 controls		36.6	29.5								
Panon (2019)	Thailand	67 overweight	Single	47	49.3	Age/sex	Zeiss Cirrus HD	+	-	+	-	+	+
		53 controls		45.6	35.9								
Laiginhas (2019)	Portugal	36 morbid and super obese	Mixed	49	14	Age	Heidelberg Spectralis	-	-	-	+	-	-
		20 controls		53	25								
Baran (2019)	Turkey	58 obese	Both	14.7	34	-	Zeiss Cirrus HD	+*	-	-	+	+	+
		35 controls		15.5	43								
Topcu-Yilmaz (2018)	Turkey	19 obese	Single	10.7	47.4	Age/sex	Heidelberg Spectralis	-	-	-	-	-	+
		26 controls		11	57.7								
Özen (2018)	Turkey	38 obese	Both	12.8	47.4	-	Heidelberg Spectralis	-	-	+	-	-	-
		40 controls		12.9	52.5								
Öner (2018)	Turkey	32 obese	N/A	41.9	25	-	Heidelberg Spectralis	-	-	-	-	-	+
		45 controls		N/A	N/A								
Koca (2017)	Turkey	63 obese	Mixed	12.9	36	-	Zeiss Cirrus HD	-	-	+	-	+	-
		62 controls		13	48								
Karti (2017)	Turkey	54 obese	Both	14.1	31.5	-	Zeiss Cirrus HD	-	+	-	+	-	-
		33 controls		13.4	38.5								
Bulus (2017)	Turkey	44 obese	Both	13.3	38.6	Age/sex	Heidelberg Spectralis	-	-	-	-	-	+
		42 controls		13.4	45.2								
Yumusak (2016)	Turkey	72 obese	Single	37.3	0	-	Nidek RS-3000	-	-	-	-	-	+
		68 controls		37.9	0								
Erşan (2016)	Turkey	40 obese	Single	11.2	42.5	Age/sex	Zeiss Cirrus HD	+	-	-	-	-	+
		40 controls		11.3	42.5								
Dogan (2016)	Turkey	67 morbid obese	N/A	37.9	N/A	Age/sex	Zeiss Cirrus HD	+	-	+	-	+	+
		29 controls		35.3	N/A								
Demir (2016)	Turkey	95 obese	Single	10.8	49.4	Age/sex	Zeiss Cirrus HD	-	+	-	+	-	-
		30 controls		11.1	53.3								
Yilmaz (2015)	Turkey	40 obese	Single	29.3	42.5	-	Heidelberg Spectralis	+	-	-	-	-	+
		40 controls		27.2	55								
Pacheco-Cervera (2015)	Spain	42 obese	Both	11	52.4	-	Zeiss Cirrus HD	-	-	-	+	-	-
		31 controls		11.1	58.1								

**Abbreviations:** SD-OCT: Spectral-domain optical coherence tomography, GC-IPL: Ganglion Cell-Inner Plexiform Layer, RNFL: Retinal Nerve Fiber Layer, pRNFL: peripapillary RNFL, N/A: Not available

* “+” refers to the SD-OCT measurements reported by the studies, whereas the “-” were not reported by the studies.

### 3.1. Macular thickness

In total, 5 studies examined macular thickness, responsible for 272 cases and 197 controls [9, 20, 26–28]. No significant difference was observed between the groups (SMD: -0.20, 95% CI: -0.61 to 0.22, p = 0.36, **S1 Fig**). We also performed a sensitivity analysis to examine what was the result of the studies that reported mean (SD), as Baran et al. data were converted to mean (SD) as mentioned in the methods. The remaining four studies also did not show a significant difference (SMD: -0.08, 95% CI: -0.53 to 0.37, p = 0.74).

### 3.2. Macular Ganglion Cell-Inner Plexiform Layer (GC-IPL) thickness

3 studies reported GC-IPL thickness, with 202 cases and 197 controls investigating temporal-superior macular sector and 180 cases and 104 controls examining the other sectors [10, 29, 30]. Studies reported the thickness of these layers together due to their similar reflectivity that made their separate detection difficult [17, 37].

We observed no changes in average GC-IPL thickness (SMD: -0.22, 95% CI: -0.47 to 0.03, p = 0.08, **S2 Fig**), and in any of the sub-sectors of macula: nasal-superior (SMD: -0.22, 95% CI: -0.46 to 0.03, p = 0.08), temporal-superior (SMD: -0.23, 95% CI: -0.53 to 0.07, p = 0.14), nasal-inferior (SMD: -0.18, 95% CI: -0.43 to 0.07, p = 0.15), temporal-inferior (SMD: -0.22, 95% CI: -0.47 to 0.02, p = 0.07), and inferior (SMD: -0.20, 95% CI: -0.45 to 0.05, p = 0.11).

### 3.3. Retinal Nerve Fiber Layer (RNFL) thickness

RNFL was investigated by 5 studies in 336 cases and 279 controls [12, 27, 28, 31, 32]: 298 cases and 239 controls in 4 studies examined average RNFL [27, 28, 31, 32] and 202 cases and 197 controls in 3 articles studied nasal and temporal RNFL [12, 31, 32]. Average (SMD: -0.13, 95% CI: -0.30 to 0.04, p = 0.13, **S3 Fig**) and nasal RNFL thickness (SMD: -0.11, 95% CI: -0.30 to 0.09, p = 0.29, **S4 Fig**) was similar between the groups, but temporal RNFL thickness (SMD: -0.33, 95% CI: -0.53 to -0.14, p<0.01, **S5 Fig**) was reduced in patients with high BMI.

### 3.4. Peripapillary Retinal Nerve Fiber Layer (pRNFL) thickness

pRNFL thickness was measured in 7 studies with 366 cases and 240 controls examining this parameter [10, 11, 13, 25, 26, 29, 30], and 5 studies with 272 cases and 185 reported detailed data on the thickness of the four quadrants [10, 13, 25, 29, 30]. Patients with higher BMI had lower average pRNFL thickness with borderline significance (SMD: -0.26, 95% CI: -0.53 to 0.00, p = 0.05, **S6 Fig**); however, none of the four quadrants had different thicknesses between the groups: superior pRNFL (SMD: -0.22, 95% CI: -0.54 to 0.11, p = 0.19), nasal pRNFL (SMD: -0.13, 95% CI: -0.47 to 0.21, p = 0.45), temporal pRNFL (SMD: -0.18, 95% CI: -0.45 to 0.10, p = 0.21), and inferior pRNFL (SMD: -0.08, 95% CI: -0.38 to 0.21, p = 0.58).

### 3.5. Cup to disc ratio

In total, 5 studies examined cup to disc ratio of 296 cases and 220 controls [26–28, 30, 31]. Cup to disc ratio was similar between the groups (SMD: -0.16, 95% CI: -0.33 to 0.02, p = 0.08, **S7 Fig**). A sensitivity analysis was performed only on the studies reporting mean (SD) [27, 28, 30] that also did not yield a significant result (SMD: 0.00, 95% CI: -0.23 to 0.24, p = 1.00).

### 3.6. Choroidal thickness

A total of 12 studies measured various parameters of choroidal thickness, accounting for 634 cases and 569 controls [9, 14, 20, 25–28, 32–36]. Average choroidal thickness was not statistically different between cases and controls (SMD: 0.27, 95% CI: -0.18 to 0.72, p = 0.24, **S8 Fig**).

We also observed no significant different thickness in the following regions: 0.5 mm nasal to fovea (SMD: -0.06, 95% CI: -0.38 to 0.25, p = 0.70), 0.5 mm temporal to fovea (SMD: -0.05, 95% CI: -0.49 to 0.38, p = 0.81), 1.5 mm nasal to fovea (SMD: -0.14, 95% CI: -0.40 to 0.12, p = 0.29), and 1.5 mm temporal to fovea (SMD: -0.17, 95% CI: -0.60 to 0.26, p = 0.44). However, the sub-foveal region (SMD: -0.24, 95% CI: -0.49 to 0.00, p = 0.05, **S9 Fig**), the region 1.0 mm nasal to fovea (SMD: -0.38, 95% CI: -0.60 to -0.16, p<0.01, **S10 Fig**), and the region 1.0 mm temporal to fovea (SMD: -0.38, 95% CI: -0.75 to 0.00, p = 0.05, **S11 Fig**) had significantly or borderline decreased thickness. **Table 2** illustrates the analyses for all the SD-OCT measurements.

**Table 2:** Comparing ocular layers between patients with higher BMI and normal weight patients based on SD-OCT measurements.

SD-OCT measurement	Overall effect		Heterogeneity	
	SMD (95% CI)	p-value	I^2^ (%)	Q (P)
**Macular thickness**	-0.20 [-0.61, 0.22]	0.36	80%*	<0.01
**Macular GC-IPL thickness**				
Average	-0.22 [-0.47, 0.03]	0.08	0%	0.42
Nasal-superior	-0.22 [-0.46, 0.03]	0.08	8%	0.34
Temporal-superior	-0.23 [-0.53, 0.07]	0.14	54%*	0.11
Nasal-inferior	-0.18 [-0.43, 0.07]	0.15	0%	0.40
Temporal-inferior	-0.22 [-0.47, 0.02]	0.07	0%	0.69
Inferior	-0.20 [-0.45, 0.05]	0.11	0%	0.39
**RNFL thickness**				
Average	-0.13 [-0.30, 0.04]	0.13	0%	0.41
Nasal	-0.11 [-0.30, 0.09]	0.29	0%	0.99
Temporal	-0.33 [-0.53, -0.14]	<0.01*	4%	0.35
**Peripapillary RNFL thickness**				
Average	-0.26 [-0.53, 0.00]	0.05*	61%*	0.02
Superior	-0.22 [-0.54, 0.11]	0.19	65%*	0.02
Nasal	-0.13 [-0.47, 0.21]	0.45	68%*	0.01
Temporal	-0.18 [-0.45, 0.10]	0.21	53%*	0.08
Inferior	-0.08 [-0.38, 0.21]	0.58	58%*	0.05
**Cup to disc ratio**	-0.16 [-0.33, 0.02]	0.08	32%	0.21
**Choroidal thickness**				
Average	0.27 [-0.18, 0.72]	0.24	68%*	0.04
Sub-foveal region	-0.24 [-0.49, 0.00]	0.05*	65%*	<0.01
0.5 mm nasal to fovea	-0.06 [0.38, 0.25]	0.70	69%*	0.01
0.5 mm temporal to fovea	-0.05 [-0.49, 0.38]	0.81	84%*	<0.01
1.0 mm nasal to fovea	-0.38 [-0.60, -0.16]	<0.01*	0%	0.38
1.0 mm temporal to fovea	-0.38 [-0.75, 0.00]	0.05*	57%*	0.11
1.5 mm nasal to fovea	-0.14 [-0.40, 0.12]	0.29	54%*	0.06
1.5 mm temporal to fovea	-0.17 [-0.60, 0.26]	0.44	83%*	<0.01

Significant or borderline p-values of 0.05 or less for overall effect and I^2^>40% are marked in bold (*).

**Abbreviations:** SD-OCT: Spectral-domain optical coherence tomography, SMD: Standardized mean difference, CI: Confidence interval, GC-IPL: Ganglion Cell-Inner Plexiform Layer, RNFL: Retinal Nerve Fiber Layer, pRNFL: peripapillary RNFL

We also performed a sensitivity analysis for sub-foveal region thickness including the studies that reported their data as mean (SD), and found no significant difference between sub-foveal thickness of cases and controls (SMD: -0.20, 95% CI: -0.46 to 0.06, p = 0.13).

### 3.7. Meta-regression

We conducted meta-regression on 7 studies comprising average pRNFL and 9 studies on sub-foveal choroidal region thicknesses. Number of studies on other measurements were not sufficient for performing meta-regression. We observed significant associations between average pRNFL thickness and male proportion (β = 0.0097, p = 0.016, r^2^ = 72.49%), mean spherical equivalents of controls (β = -4.2076, p = 0.007, r^2^ = 100.00%), body mass index standard deviation score (BMISDS) of controls (β = -5.1802, p = 0.020, r^2^ = 100.00%), and Homeostatic model assessment for Insulin resistance (HOMA-IR) of cases (β = -0.3536, p = 0.024, r^2^ = 100%), while sub-foveal choroidal thickness was only associated with mean intraocular pressure (IOP) of cases (β = -0.1584, p = 0.017, r^2^ = 65.48%).

Insignificant variables for both measurements included study sample size, year of study, mean age, matching status for age/sex, eye measurement method (single-eye vs. both eyes), OCT models, mean intraocular pressure of controls, mean Spherical equivalent of cases, and body mass index (BMI) of cases. Insignificant variables for either of average pRNFL and sub-foveal thicknesses—significant or unavailable data for the other- were the male proportion, mean intraocular pressure of cases, mean Spherical equivalent of controls, ocular axial length of cases and controls, BMI of controls, BMISDS of controls, triglyceride level of cases and controls, cholesterol level of cases, low density lipoprotein (LDL) level of cases, high density lipoprotein (HDL) of cases, HOMA-IR of controls, systolic blood pressure (SBP) of cases and controls, and diastolic blood pressure (DBP) of cases and controls (**Table 3**).

**Table 3:** Detailed results of performed meta-regression.

Variables	Average pRNFL thickness		Sub-foveal choroidal thickness	
	β coefficient	p-value	β coefficient	p-value
Study sample size	0.0077	0.329	0.0018	0.808
Year of study	0.0005	0.995	0.0060	0.936
Mean age	-0.0027	0.821	0.0031	0.772
Male proportion	0.0097	0.016*	-0.0044	0.400
Matching status for age/sex	0.0422	0.889	0.0471	0.859
Eye measurement method (Single or both eyes)	-0.0583	0.725	0.0722	0.805
OCT models	0.2150	0.211	-0.2952	0.065
Mean intraocular pressure- cases	0.0614	0.523	-0.1584	0.017*
Mean intraocular pressure- controls	0.0424	0.709	-0.0983	0.484
Mean Spherical equivalent- cases	-1.5635	0.245	6.2370	0.565
Mean Spherical equivalent- controls	-4.2076	0.007*	1.4700	0.628
Ocular axial length- cases	N/A	N/A	-0.1092	0.847
Ocular axial length- controls	N/A	N/A	0.2028	0.762
BMI- cases	-0.0047	0.931	-0.0215	0.296
BMI-controls	N/A	N/A	-0.4892	0.580
BMISDS- controls	-5.1802	0.020*	N/A	N/A
Triglyceride level- cases	-0.0042	0.107	N/A	N/A
Triglyceride level- controls	-0.0063	0.141	N/A	N/A
Cholesterol level- cases	0.0058	0.908	N/A	N/A
LDL level- cases	-0.0290	0.864	N/A	N/A
HDL level- cases	-0.0110	0.141	N/A	N/A
HOMA-IR- cases	-0.3536	0.024*	N/A	N/A
HOMA-IR-controls	-0.6755	0.501	N/A	N/A
Systolic blood pressure- cases	N/A	N/A	0.0031	0.912
Systolic blood pressure- controls	N/A	N/A	-0.0084	0.758
Diastolic blood pressure- cases	N/A	N/A	0.0156	0.610
Diastolic blood pressure- controls	N/A	N/A	0.0038	0.917

Significant p-values of less than 0.05 are marked in bold (*).

**Abbreviations:** pRNFL: Peripapillary Retinal Nerve Fiber Layer, OCT: Optical coherence Tomography, N/A: Not available, BMI: Body mass index, BMISDS: Body mass index standard deviation score, LDL: Low density lipoprotein, HDL: High density lipoprotein, HOMA-IR: Homeostatic model assessment for Insulin resistance

### 3.8. Subgroup analysis

We performed subgroup analysis on the average pRNFL and sub-foveal choroidal region thicknesses, as they had sufficient number of studies. Both parameters had borderline significance (p = 0.05) as mentioned above. **Table 4** presents the details of the sub-groups analyses for these two parameters.

**Table 4:** Detailed results of sub-group analyses for the average pRNFL thickness and sub-foveal choroidal thickness.

SD-OCT measurement	Number of studies	Overall effect	
		SMD (95% CI)	p-value
**1. Average pRNFL thickness**	7	-0.26 [-0.53, 0.00]	0.05*
Adults	2	-0.11 [-0.77, 0.55]	0.63
Children	5	-0.33 [-0.63, -0.03]	0.03*
Single eye datasets	2	-0.14 [-0.44, 0.16]	0.37
Both eyes datasets	4	-0.29 [-0.74, 0.17]	0.21
Mixed eyes datasets	1	-0.48 [-1.03, 0.07]	0.09
Studies matched for age/sex	3	-0.22 [-0.48, 0.04]	0.10
Studies not matched for age/sex	4	-0.29 [-0.74, 0.17]	0.21
Zeiss Cirrus HD	5	-0.33 [-0.63, -0.03]	0.03*
Heidelberg spectralis	1	-0.48 [-1.03, 0.07]	0.09
3D OCT-2000 FA plus	1	0.20 [-0.19, 0.59]	0.27
**2. Sub-foveal choroidal thickness**	9	-0.24 [-0.49, 0.00]	0.05*
Adults	5	-0.23 [-0.44, -0.02]	0.03*
Children	4	-0.26 [-0.80, 0.27]	0.25
Single eye datasets	5	-0.21 [-0.43, 0.00]	0.05*
Both eyes datasets	3	-0.16 [-0.82, 0.51]	0.65
Studies matched for age/sex	5	-0.23 [-0.64, 0.19]	0.28
Studies not matched for age/sex	4	-0.27 [-0.54, -0.01]	0.04*
Zeiss Cirrus HD	4	-0.43 [-0.69, -0.18]	<0.01*
Heidelberg spectralis	4	0.02 [-0.38, 0.41]	0.93
3D OCT-2000 FA plus	1	-0.42 [-0.81, -0.02]	0.04*

Significant p-values of less than 0.05 are marked in bold (*).

**Abbreviations:** SD-OCT: Spectral-domain optical coherence tomography, SMD: Standardized mean difference, CI: Confidence interval, pRNFL: peripapillary Retinal Nerve Fiber Layer

#### 3.8.1. Average pRNFL

We found that the average pRNFL thickness was only statistically lower in higher BMI children (SMD: -0.33, 95% CI: -0.63 to -0.03, p = 0.03, **S12 Fig**) and the studies utilizing Zeiss Cirrus HD (SMD: -0.33, 95% CI: -0.63 to -0.03, p = 0.03), both comprising identical studies. Subgroup analysis for average pRNFL thickness was non-significant for adults (**S12 Fig**), single eye datasets, both eyes datasets, mixed eyes datasets (**S13 Fig**), and studies matched for age/sex and those not matched for them (**S14 Fig**). Heidelberg spectralis and 3D OCT-2000 FA plus each had 1 study that were also non-significant.

#### 3.8.2. Sub-foveal region

The difference in sub-foveal thickness was repeated only in the studies not age/sex matched (SMD: -0.27, 95% CI: -0.54 to -0.01, p = 0.04), and the matched studies showed non-significant results (p = 0.28, **S15 Fig**). Contrary to the average pRNFL, sub-foveal thickness was thinner in high BMI adults (SMD: -0.23, 95% CI: -0.44 to -0.02, p = 0.03), while no relationship was established in children (p = 0.25, **S16 Fig**). Studies utilizing Zeiss Cirrus HD (SMD: -0.43, 95% CI: -0.69 to -0.18, p<0.01) and only 1 study using 3D OCT-2000 FA plus (SMD: -0.42, 95% CI: -0.81 to -0.02, p = 0.04) had significantly different thicknesses, while such relationship was not observed for Heidelberg spectralis (**S17 Fig**). The results had borderline significance in single eye datasets (SMD: -0.21, 95% CI: -0.43 to 0.00, p = 0.05), but was not significant in studies measuring both eyes (p = 0.65, **S18 Fig**).

### 3.9. Publication bias

We performed Egger’s test for all the analyzes and found no publication bias in any of them, except for the macular thickness and average choroidal thickness that had significant Egger’s test and asymmetrical funnel plot. Trim and fill was executed for these two parameters. The analysis for average choroidal thickness remained the same before and after trim and fill. Trim and fill method calculated corrected numbers for macular thickness that confirmed the observed insignificant relationship between the BMI and macular thickness (SMD: -0.08, 95% CI: -0.49 to 0.33, p = 0.72) (**S2 Table**).

### 3.10. Quality assessment

Most of the studies scored acceptable to good scores based on the NOS (mean: 5.5, range: 3–8, out of 8). **S1 Table** illustrates the quality score details based on their scores in selection, comparability, and exposure. The least favorable component was their comparability, as many studies did not match their cases and controls with each other regarding age or sex. However, most of studies scored well in the selection and comparability categories.

## 4. Discussion

In this meta-analysis, we found that most of the SD-OCT measurements trended towards decreased thickness, but only two measurements were significantly thinner in patients with high BMI compared to controls; temporal RNFL and the choroidal region 1.0 mm nasal to fovea (p<0.01 for both). Three other measurements had borderline significance (p = 0.05): average pRNFL, sub-foveal choroidal thickness, and the choroidal region 1.0 mm temporal to fovea.

Several mechanisms can explain the global RNFL thinning that was only significant in temporal RNFL and average pRNFL. First, neurological consequences of obesity might partly play a role in these findings [38]. Patients with obesity are at increased risk for neurological disorders, such as Parkinson’s, Alzheimer’s disease, and other types of dementia [39, 40]. The increased risk can be attributed to the excessive susceptibility of neuronal cells to oxidative stress cause by low-grade inflammation in obesity and the changed ubiquitin-proteasome pathway [41, 42]. Some neurological diseases, including Alzheimer’s disease, might cause retrograde optic nerve degeneration, as well as simultaneous direct retinal involvements per se [17, 43]. Reduced lipoprotein lipase activity and visceral fat accumulation were introduced as some of the factors inducing neurodegenerative retinal disorders [43]. Therefore, it is suggested that RNFL measurements can serve as a marker of systemic neuro-degeneration in the patients with obesity [44].

Aging can be another contributing factor and we can divide it into actual and accelerated aging mechanisms. RNFL thickness decreases after 50 years of age, by 2.2 μm per decade [45, 46]. The prevalence of obesity increases with aging until the age of 50–59 when it reaches its peak prevalence [47]. Therefore, studies unadjusted for age can produce lower RNFL thicknesses for patients with obesity partly due to their older age, although such hypothesis can only be applicable for 50–59 years when obesity prevalence increases and RNFL thickness decreases. However, this might not be the case as we found no relationship between mean age and average pRNFL thickness in meta-regression, and age-matched and non-age-matched subgroup analyses did not produce different results. Furthermore, our sub-group analysis yielded significant results only for children and not the adults. This shows that the significantly lower average pRNFL thickness came from the significance of the studies on children, and not the adults. Therefore, the unmatched studies on adults could not confound the significantly lower average pRNFL thickness due to the confounder of higher obesity prevalence and lower average pRNFL thickness in people older than 50. On the other hand, obesity can accelerate the epigenetic aging process [48]. Therefore, it is possible that RNFL thinning can be started earlier in the life of the patients with obesity due to their premature aging. Premature aging may also contribute the significantly lower average pRNFL thickness in children.

Increased IOP might be another factor of decreased RNFL in obesity. Several previous studies established a relationship between obesity and increased IOP [26, 28, 49]. One study considered their statistically significant increase too small to be of clinical significance [26]. Glaucoma and increased IOP might play roles in RNFL thinning [50–52]. However, a large population-based study of 11,030 healthy eyes found that IOP was not associated with any RNFL measurements; but the authors attributed this finding to the lack of data on the pre-treatment pressures of the individuals on IOP-lowering therapies [53]. A recent population-based study demonstrated that after adjusting for the potential confounders, each 1 mmHg increase in the IOP correlated with a 0.05 μm/year faster decrease in RNFL thickness [54]. We did not observe such correlations between IOP of cases and controls and average pRNFL thickness. However, other specific sub-sectors could have such correlations that we could not analyze due to the lack of sufficient studies.

Obesity induces a low-grade inflammatory status in the body [55]. Increased mitochondrial and peroxysomal fatty acid oxidation leads to higher oxidative stress, thereby inducing systemic inflammation [56]. Excessive lipid mass increases secretion of pro-inflammatory cytokines and adipokines such as leptin, resistin, IL-6, tumor necrosis factor-alpha (TNF-α), and plasminogen activator inhibitor-1 (PAI-I), and suppresses the anti-inflammatory cytokines such as adiponectin [26, 57, 58]. Markers of endothelial activation and dysfunction are also increased in individuals with obesity [10]. Leptin, for instance, is a hormone secreted by adipose tissue that regulates energy intake and expenditure and serves as an acute-phase reactant [59]. It has similar functional and structural similarities to IL-6 and play roles in differentiation, proliferation, and survival of numerous cells including endothelial cells, lymphocytes, and neutrophils, reducing vasodilator response by nitric oxide (NO), and increasing oxidative stress [57, 60]. The adipokines then provoke the production of reactive oxygen species (ROS) and promote the level of oxidative stress [61]. Endothelial dysfunction in obesity leads to decreased vasodilator molecules, such as NO, and increased levels of vasoconstrictor compounds, again stimulating inflammation and ROS production [57, 62]. The ongoing inflammation and increased ROS promote ganglion cell death and axonal loss following axonal injury via both apoptosis and necrosis pathways [56]. Therefore, inflammatory pathways in obesity lead to a decreased RNFL, in fact, they are also responsible for some of the other abovementioned mechanisms, including neurological disorders, aging, and increased IOP [11, 25, 28].

The last mechanism involves other metabolic disorders and hypertension coexisting with obesity. Although we did not observe a relationship between lipid profile and average pRNFL thickness in the meta-regression, HOMA-IR and BMISDS reduced its thickness. Zarei et al. previously established the relationship between higher number of metabolic abnormalities and lower RNFL thickness [48]. Chronic inflammation and increased oxidative stress stimulate insulin resistance and also deprive retinal cells of neurotrophic factors essential for their survival, including insulin, leukemia inhibitory factor, and ciliary neurotrophic factor [63]. Baran et al. also observed a negative correlation between BMISDS and waist-hip ratio (WHR)- representing central obesity- with decreased RNFL thickness [26]. Hypertension was another factor that we could not examine in the meta-regression, but was previously introduced as a possible factor responsible for RNFL thinning. Hypertension seemed to affect the temporal RNFL quadrant more than the others that can partly explain why we observed significant RNFL thinning only in this quadrant [64]. Overall, we should holistically take these proposed mechanisms for RNFL thinning into account, as they are related to each other and not isolated pathways.

Male proportion, mean spherical equivalent, BMISDS, and HOMA-IR had correlations with average pRNFL in meta-regression. Previous literature also suggested such correlations. Epic-Norfolk study found thinner RNFL in older patients, male sex, higher BMI (only in males), short axial length, and the history of cataract surgery, but not an increased IOP [53]. Refractive status also affected RNFL measurements in previous studies [65, 66]. As mentioned above, insulin is considered a neurotrophic factor essential for retinal cell survival, and therefore, insulin resistance measured by HOMA-IR contributes to lower average pRNFL thickness [10, 63]. The correlation between BMISDS and decreased RNFL thickness was observed in other studies [12, 26]. We did not find correlations between age and average pRNFL thickness. This could be related to the included studies, as most of them recruited children. Children and adolescents of various ages have similar RNFL thickness [67, 68], and the decrease in RNFL thickness starts around 50 years old [46]. Therefore, we expect that our sample population could not show such correlation, even if it existed.

We observed that sub-foveal choroidal thickness, the choroidal region 1.0 mm nasal to fovea, and the choroidal region 1.0 mm temporal to fovea were significantly thinner in patients with higher BMI. Choroid is the vascular layer of the eye and 65–85% of the ocular blood flow passes through its vascular plexus [35]. Higher BMI is associated with structural changes in the macrovascular and microvascular system [19, 69]. Therefore, the observed changes in the choroidal vasculature might be responsible for the observed decrease in the choroidal thickness [36].

Obesity provokes changes in the molecular environment of choroid and subsequently causes changes in the diameter of the retinal vasculature. Choroid is the only layer of the eye that its vessels are regulated through sympathetic and parasympathetic mechanisms, the former reducing the choroidal blood flow with noradrenaline and the later increasing it through NO pathway [70]. Higher BMI increases the levels of vasoconstrictor molecules such as angiotensin-II and endothelin-1, and reduces the levels of vasodilators such as NO [71, 72]. NO regulates ocular perfusion to the choroidal tissue, optic nerve, and retina [73]. Retinal-originated dopamine, which secrets choroidal-originated NO, is also decreased in individuals with obesity [74, 75]. The decreased NO and dopamine levels in the eye can increase choroidal vascular resistance, reduce the blood flow, and finally decrease the choroidal thickness [75–77]. Furthermore, several studies found that obese children and adults have decreased retinal arteriolar caliber and dilated retinal venules, probably due to the dysregulations in vasodilator/vasoconstrictor balance [18, 19]. Abnormal blood flow might result from the disrupted arterial blood supply, autonomic vasoregulation, and endothelial injury, further damaging the choroid and altering its thickness [78].

We demonstrated that IOP correlated with sub-foveal choroidal thickness. As mentioned earlier, individuals with obesity have higher IOP [26, 28, 49]. Several mechanisms are linked to the higher IOP, including: 1) Increased retrobulbar fat diminishes aqueous outflow and results in a higher IOP [79], 2) Alterations in plasma levels of ghrelin and leptin can increase IOP and induce glaucoma [80, 81]. Hyperleptinemia in patients with obesity may increase the oxidative stress and spark the pathological changes causing higher IOP [81, 82]. Ghrelin is a hormone with antioxidant and anti-inflammatory effects and may affect the anterior and posterior segments of the eye [83]. Katsanos et al. found lower ghrelin levels in the anterior chamber of the glaucoma group compared to the controls [80]. Ghrelin also possessed neuro-protective properties on the retina of the glaucomatous rats [84]. Therefore, decreased ghrelin levels in patients with obesity may trigger higher IOP [83, 85]. 3) Elevated blood viscosity might increase episcleral venous pressure, hampering aqueous outflow and leading to a higher IOP [86], 4) Concurrent hyperglycemia can osmotically shift fluid into the eyes and increasing its pressure [87], 5) Altered microcirculation is another factor that increases IOP in patients with higher BMI [19]. IOP elevates in obesity partly because of the decreased levels of vasodilators, such as NO, and increased vasoconstrictors, including angiotensin-II and endothelin-I [71, 72].

Decreased choroidal thickness have been reported in several other conditions coexisting with obesity, including diabetes [88], hypertension [89], and obstructive sleep apnea-hypopnea syndrome [90]. Nevertheless, we did not find correlations between systolic and diastolic blood pressures and sub-foveal choroidal thickness. These comorbidities may be another factor affecting the choroidal thickness in patients with obesity. Fortunately, there are strategies to counter the effect of obesity on the choroidal thickness and restore ocular blood flow. Studies found that training [91] and bariatric surgery [92] improved the decreased retinal arteriole to venule ratio in obesity. Furthermore, choroidal thickness increased in the bariatric surgery group compared to those scheduled for conservative management [92].

Choroidal thickness increases in children as they get older [93, 94], and decreases after 60 years of age by 5.40 μm for each year [95]. Yumusak et al. also proposed female sex as a predictor of diminished choroidal thickness [14], but we did not observe such relationship between age and sex in sub-foveal thickness. Mapelli et al. found that children with decreased axial length had thinner choroidal volume [94], again a finding that our study did not reproduce based on sub-foveal thickness meta-regression.

We did not observe any significant changes in the GC-IPL layer thickness. This finding contrasted our expectations, as increased oxidative stress and IOP can induce ganglion cell death and reduce the GC-IPL thickness [56, 96]. Levin et al. suggested that ganglion cell death is a delayed process following the axonal injury, and many ganglion cells may survive several days after the axonal injury [96]. Factors favoring ganglion cell survival may play role in this observation [10]. Therefore, these insults may decrease GC-IPL thickness at a later stage of life. As all the included studies on GC-IPL thickness in this meta-analysis comprised children, we hypothesize that GC-IPL thickness might be decreased in the adults with higher BMI. Furthermore, low number of studies with small populations reporting these parameters might also be another factor influencing this lack of significant results, as average, nasal-superior, and temporal-inferior GC-IPL thicknesses had p-values between 0.05 and 0.1.

We did not observe a significant difference in cup to disc ratio between patients with high BMI and controls, although the p-value approached the threshold of significance (p = 0.08). Three large population-based studies found lower cup to disc ratio in patients with higher BMI [97–99]. However, none of them included pediatric patients, and two of them included patients older than 40 [97] and 45 years [98]. On the other hand, 3/5 of our studies were conducted in children [26, 30, 31] and the other two on adults [27, 28] also did not have similar compositions to the population-based studies. Therefore, differences in participants might justify the conflicting findings between this study and the aforementioned population-based studies.

Understanding the SD-OCT measurements not only impact the ocular health of the individuals with obesity, but also may have screening and prognosticating benefits for them. For instance, choroidal thickness is an important factor as it can predict several systemic and ocular complications related to metabolic syndrome, such as hypertension, diabetic retinopathy, age-related macular degeneration, and myopia [14, 18, 27, 89]. It also provides valuable non-invasive information on the important vascular changes in obesity, including cardiovascular diseases [18–20]. Changes in retinal vasculature is also known to be a marker of systemic microvascular changes in obesity [20, 34]. Retinal arteriole to venule ratio is considered an indicator of preclinical alterations in coronary and cerebral microcirculation [92]. Ganglion cell layer (GCL) thickness also correlates well with the visual field analysis and the risk of several neuro-ophthalmic pathologies in the future, including Parkinson’s and Alzheimer’s disease, which are higher among individuals with obesity [17, 100]. Therefore, SD-OCT measurements may provide precious prognostic information on the health status of the individuals with obesity, and we encourage conducting future studies and developing guidelines to detect the patients that may benefit the most from prediction ability of SD-OCT measurements. This fast, non-invasive, and accurate three-dimensional imaging may serve as an adjunct risk-stratification tool among other clinical and laboratory studies in patients with higher BMI. Nevertheless, various follow-up studies are required to carefully further assess the relationships between SD-OCT measurements and obesity, the suitability and cost-effectiveness of SD-OCT in the clinical settings, and also address the limitations of the previous studies, such as their lack of reference to/matching of various important parameters (e.g. age, sex, concurrent medications used, etc.). Furthermore, differences in results in children and adults should also be discovered in larger and more robust studies to find the best markers based on each age group.

To the best of our knowledge, this study is the first meta-analysis investigating the changes in choroidal and retinal thickness related to increased body weight. However, several limitations exist in our study. Many ocular parameters had few numbers of studies with small populations, and therefore, they may have not reached statistical significance due to this small sample size. Furthermore, studies on some parameters only consisted of specific age groups, hiding potential relationship between higher BMI and those parameters in other age groups. Another limitation arises from the countries of studies, where Turkish articles account for most of the studies. As thickness of some ocular layers vary with ethnicity, including RNFL and pRNFL [101, 102], the included studies may only represent a group of ethnicities based on their country of origin. Also, several possibly important parameters were not reported and matched for by the included studies, including the other metabolic profiles of the patients and the concurrent medications used by them. Furthermore, we could not analyze the parameters based on the severity of obesity, while some earlier studies demonstrated the degree of obesity might alter some of the findings [14].

## 5. Conclusion

Obesity is associated with diminished thickness in several SD-OCT measurements through various direct and indirect mechanisms. Although most of the layers face decreases in thickness, only two measurements reached statistical significance: temporal RNFL and the choroidal region 1.0 mm nasal to fovea. The changes in the thickness of SD-OCT measurements might serve as a risk-stratification tool to predict several ocular and systemic complications of obesity, as earlier pieces of research found associations between these complications and changes in the thicknesses of various ocular layers. Future studies and guidelines need to verify our findings, address the limitations of the studies, and establish a base for integrating SD-OCT into the care of the patients with high BMI.

## Supporting information

S1 Checklist (DOCX)

S1 Fig Comparison of macular thickness between individuals with overweight/obesity and controls. (DOCX)

S2 Fig Comparison of average GC-IPL thickness between individuals with overweight/obesity and controls. (DOCX)

S3 Fig Comparison of average RNFL thickness between individuals with overweight/obesity and controls. (DOCX)

S4 Fig Comparison of nasal RNFL thickness between individuals with overweight/obesity and controls. (DOCX)

S5 Fig Comparison of temporal RNFL thickness between individuals with overweight/obesity and controls. (DOCX)

S6 Fig Comparison of average pRNFL thickness between individuals with overweight/obesity and controls. (DOCX)

S7 Fig Comparison of cup to disc ratio between individuals with overweight/obesity and controls. (DOCX)

S8 Fig Comparison of average choroidal thickness between individuals with overweight/obesity and controls. (DOCX)

S9 Fig Comparison of the sub-foveal choroidal thickness between individuals with overweight/obesity and controls. (DOCX)

S10 Fig Comparison of the choroidal region 1.0 mm nasal to fovea thickness between individuals with overweight/obesity and controls. (DOCX)

S11 Fig Comparison of the choroidal region 1.0 mm temporal to fovea thickness between individuals with overweight/obesity and controls. (DOCX)

S12 Fig Sub-group analysis of average pRNFL thickness based on the age group of the subjects. (DOCX)

S13 Fig Sub-group analysis of average pRNFL thickness based on the method of eye selection. (DOCX)

S14 Fig Sub-group analysis of average pRNFL thickness based on the matching status of the studies. (DOCX)

S15 Fig Sub-group analysis of sub-foveal choroidal thickness based on the matching status of the studies. (DOCX)

S16 Fig Sub-group analysis of sub-foveal choroidal thickness based on the age group of the subjects. (DOCX)

S17 Fig Sub-group analysis of sub-foveal choroidal thickness based on the utilized SD-OCT model. (DOCX)

S18 Fig Sub-group analysis of sub-foveal choroidal thickness based on the method of eye selection. (DOCX)

S1 Table Trim and fill results for parameters with significant Egger’s test result. (DOCX)

S2 Table Newcastle-Ottawa Scale (NOS) risk of bias assessment of the included studies. (DOCX)
