# Supplementary figures and images for: Spectral-domain OCT measurements in obesity: A systematic review and meta-analysis
Source: PLoS One. 2022 Apr 27;17(4):e0267495. doi: 10.1371/journal.pone.0267495 (PMC9045631; doi:10.1371/journal.pone.0267495)

**
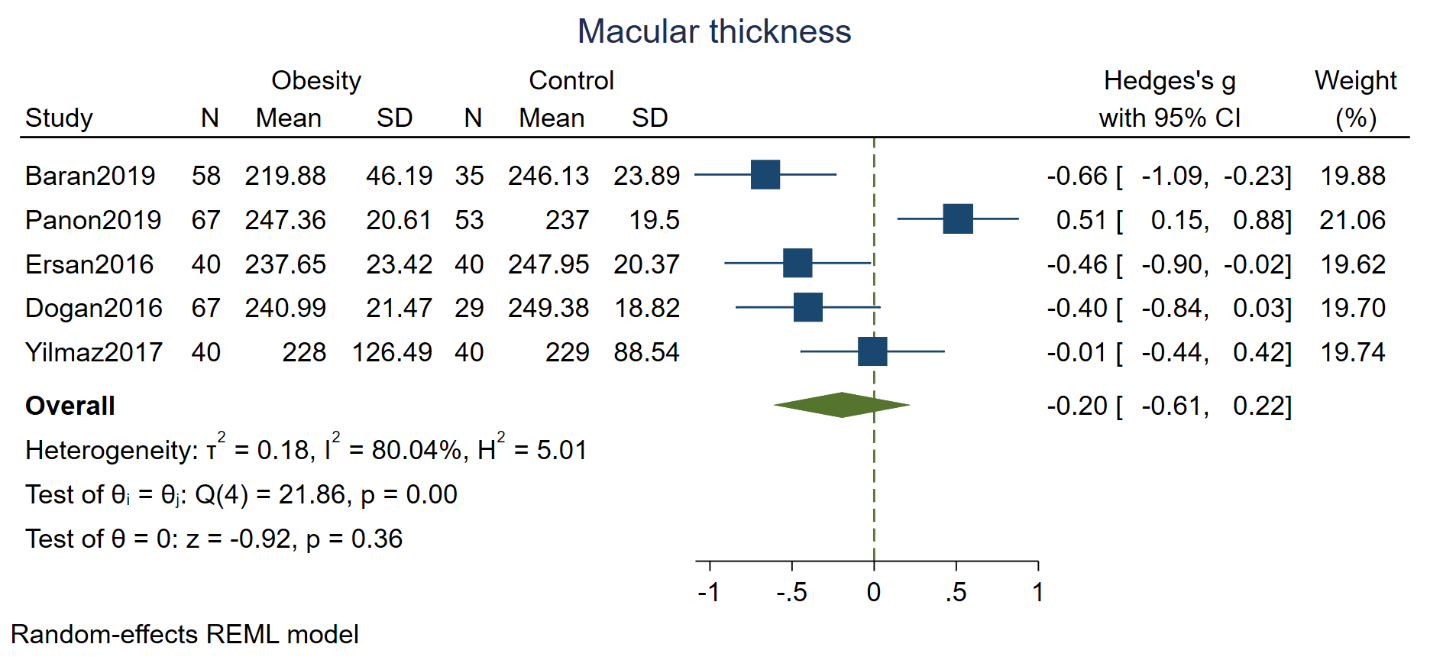
**

**S1 Fig.**

Supplement: S1 Fig — (DOCX) [file pone.0267495.s002.docx]

**
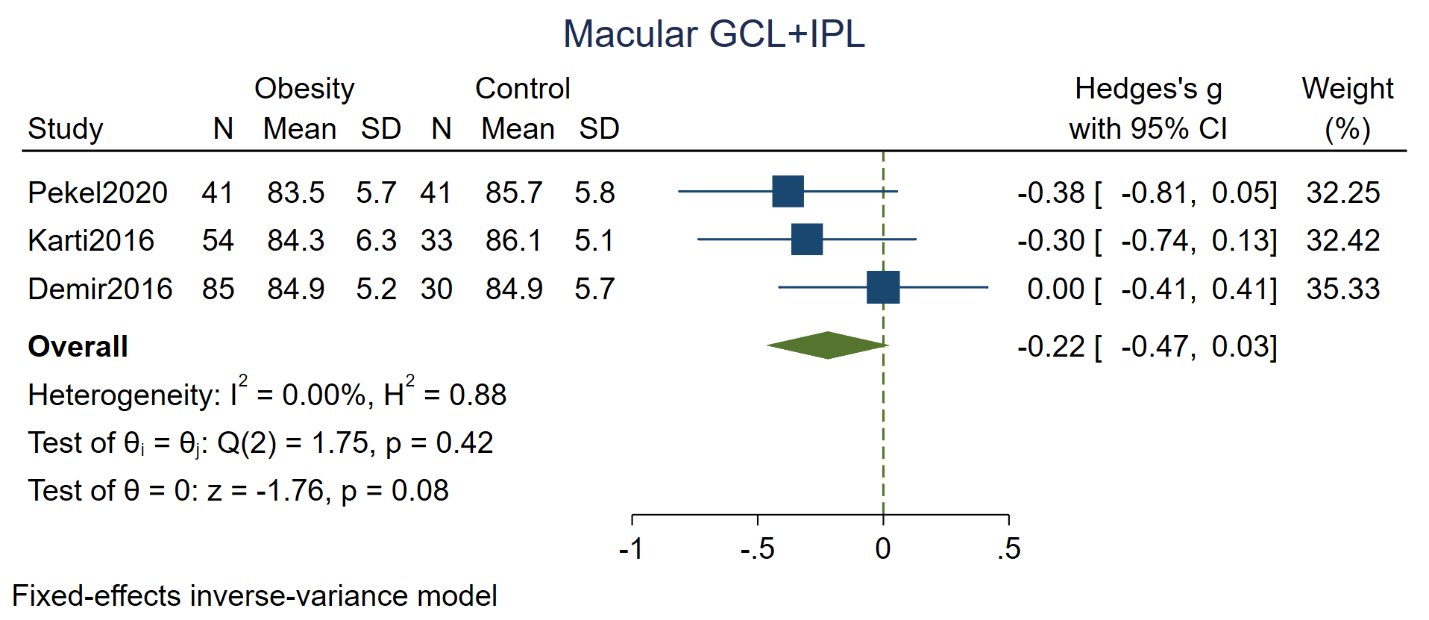
**

**S2 Fig.**

Supplement: S2 Fig — (DOCX) [file pone.0267495.s003.docx]

**
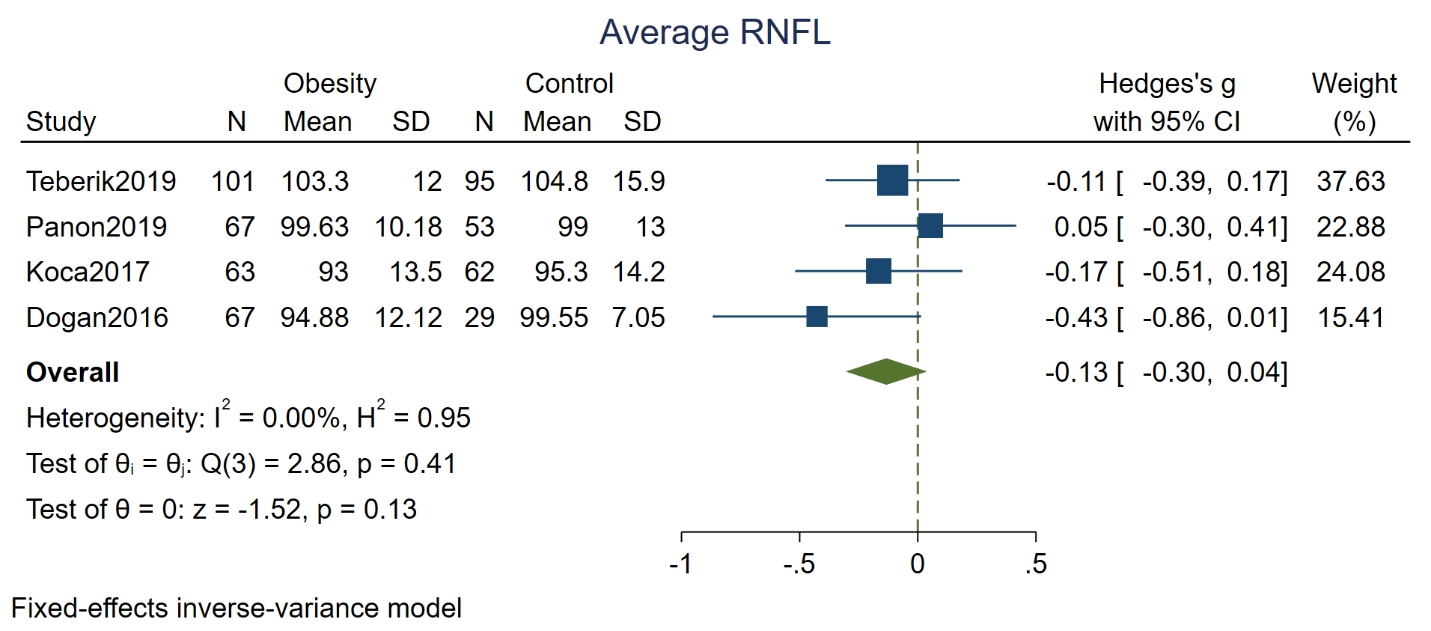
**

**S3 Fig.**

Supplement: S3 Fig — (DOCX) [file pone.0267495.s004.docx]

**
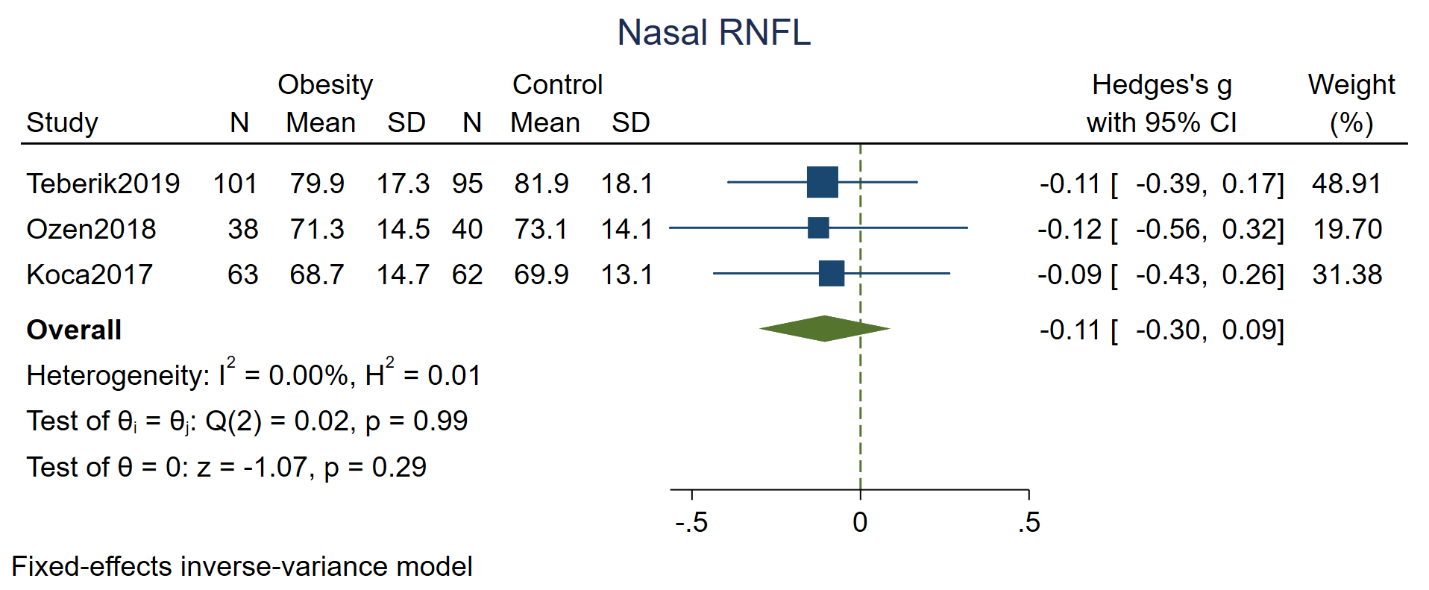
**

**S4 Fig.**

Supplement: S4 Fig — (DOCX) [file pone.0267495.s005.docx]

**
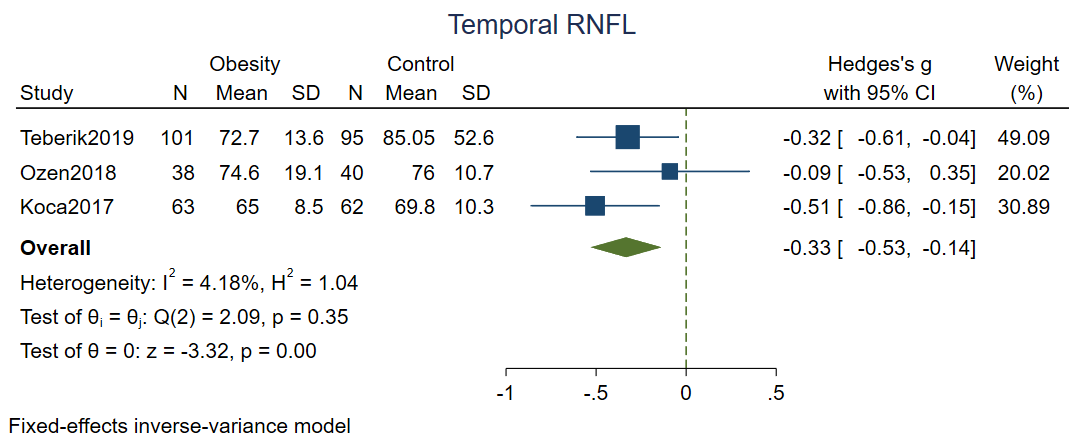
**

**S5 Fig.**

Supplement: S5 Fig — (DOCX) [file pone.0267495.s006.docx]

**
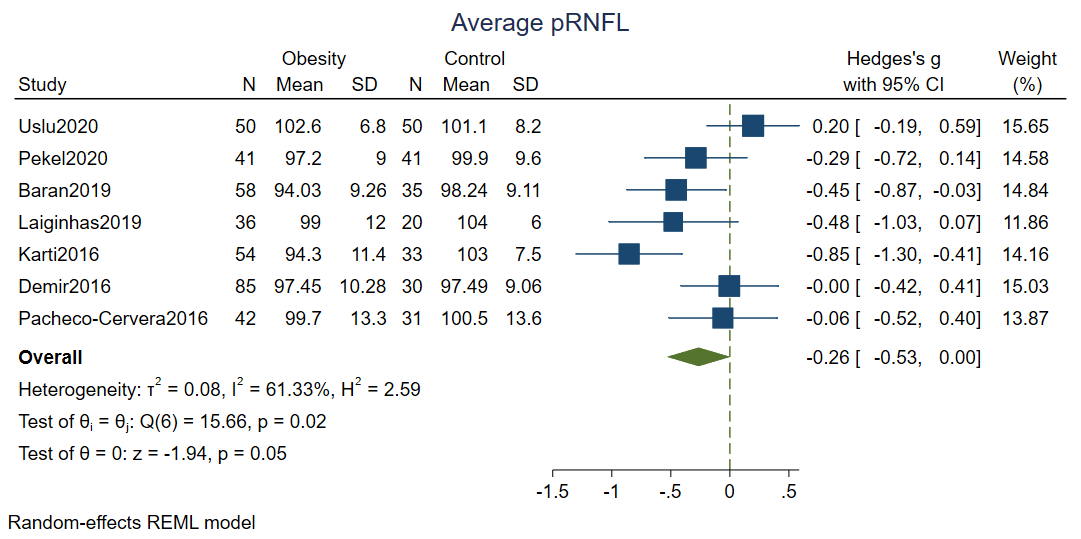
**

**S6 Fig.**

Supplement: S6 Fig — (DOCX) [file pone.0267495.s007.docx]

**
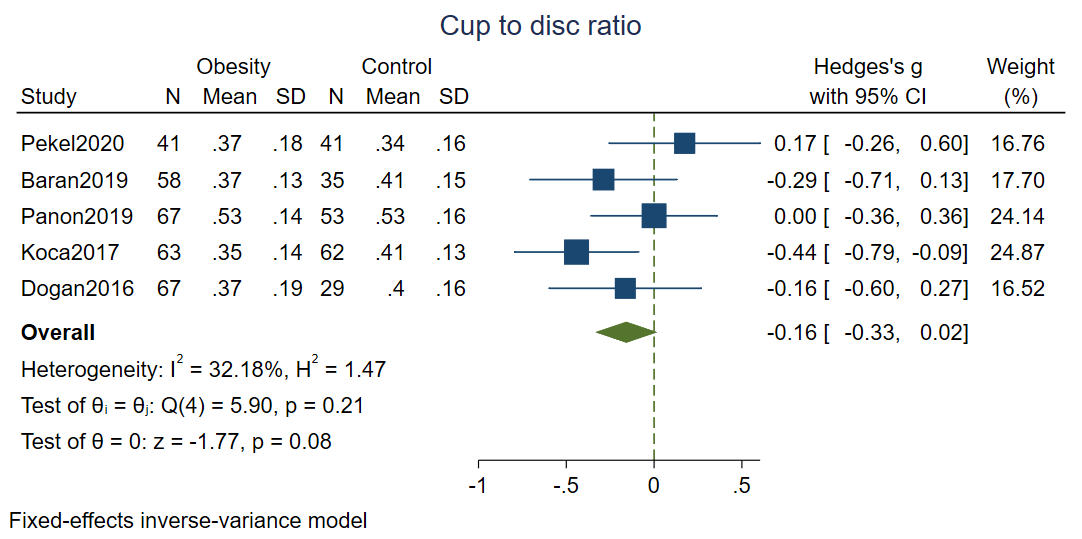
**

**S7 Fig.**

Supplement: S7 Fig — (DOCX) [file pone.0267495.s008.docx]

**
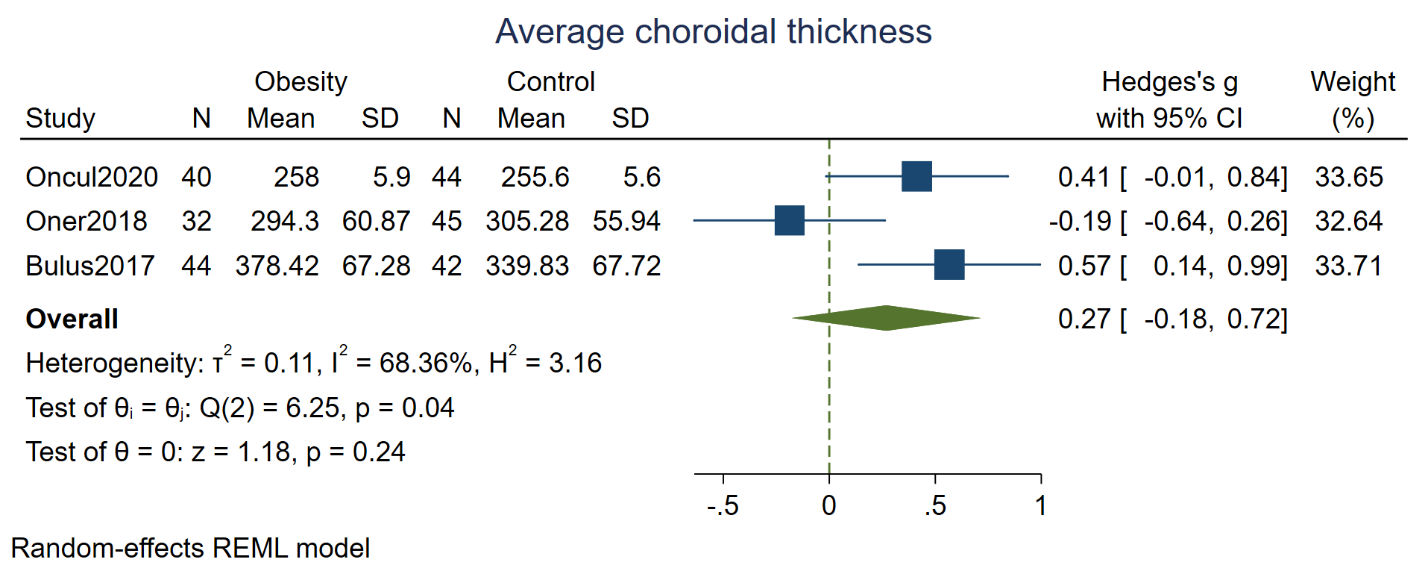
**

**S8 Fig.**

Supplement: S8 Fig — (DOCX) [file pone.0267495.s009.docx]

**
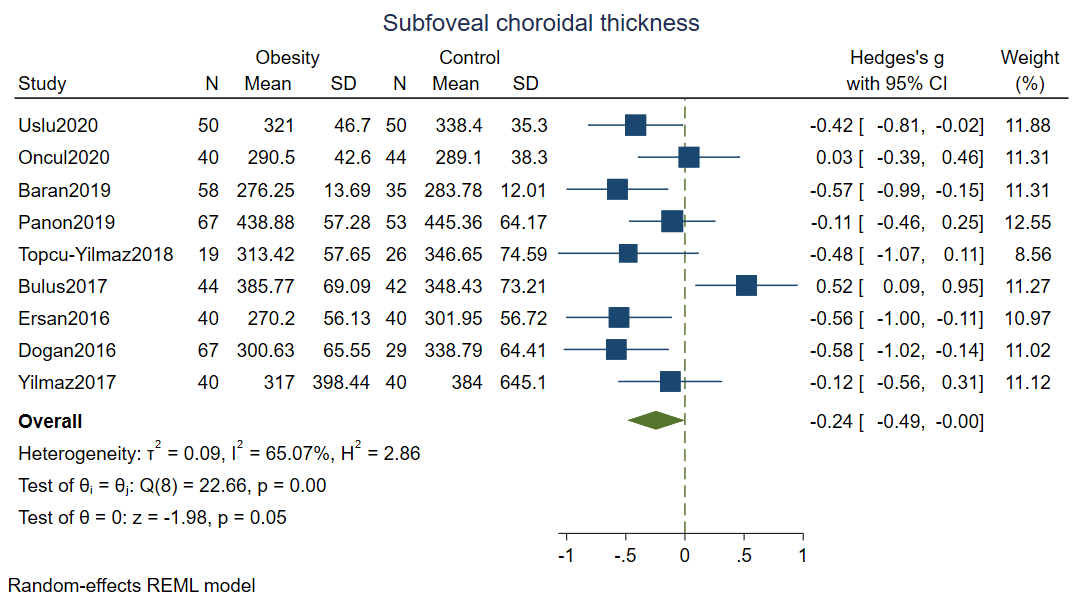
**

**S9 Fig.**

Supplement: S9 Fig — (DOCX) [file pone.0267495.s010.docx]

**
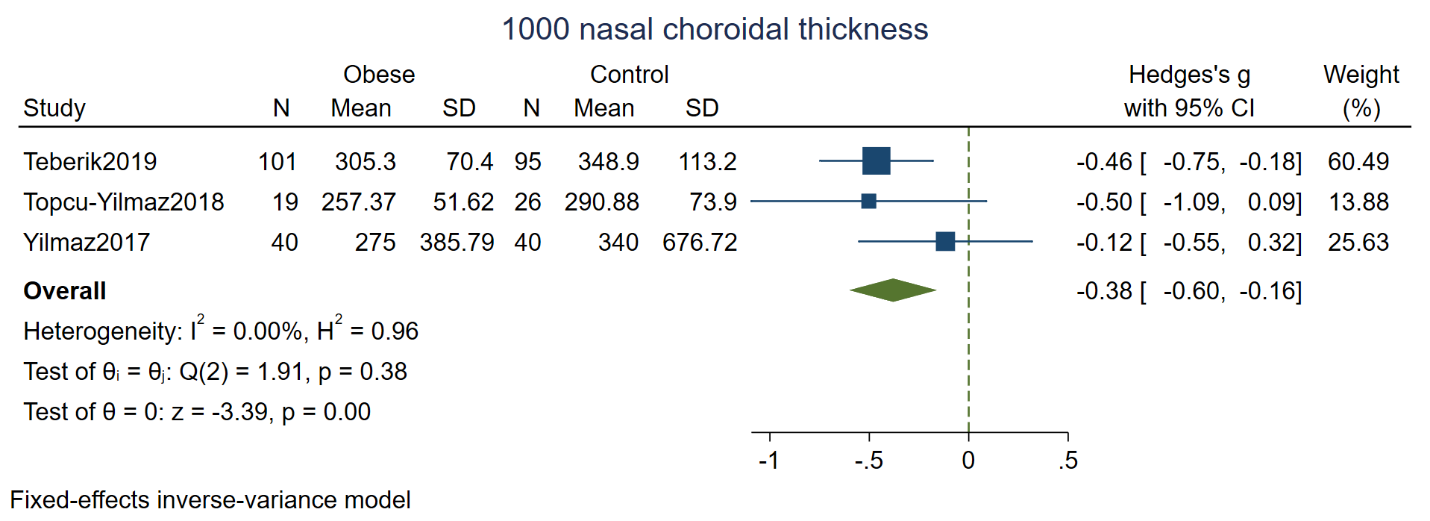
**

**S10 Fig.**

Supplement: S10 Fig — (DOCX) [file pone.0267495.s011.docx]

**
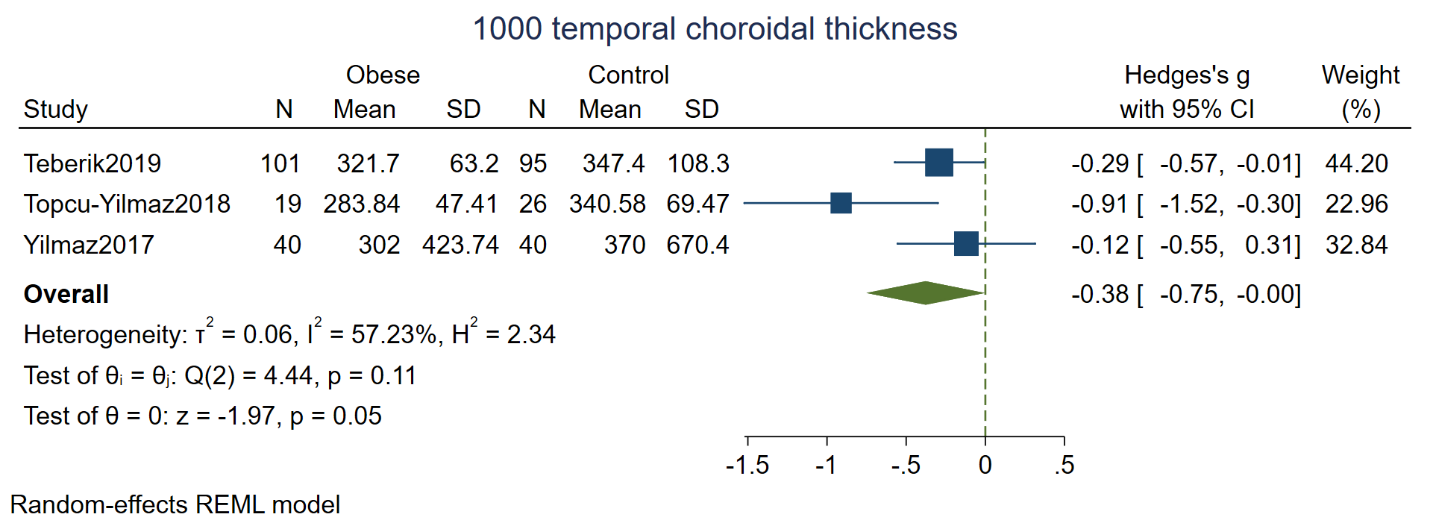
**

**S11 Fig.**

Supplement: S11 Fig — (DOCX) [file pone.0267495.s012.docx]

**
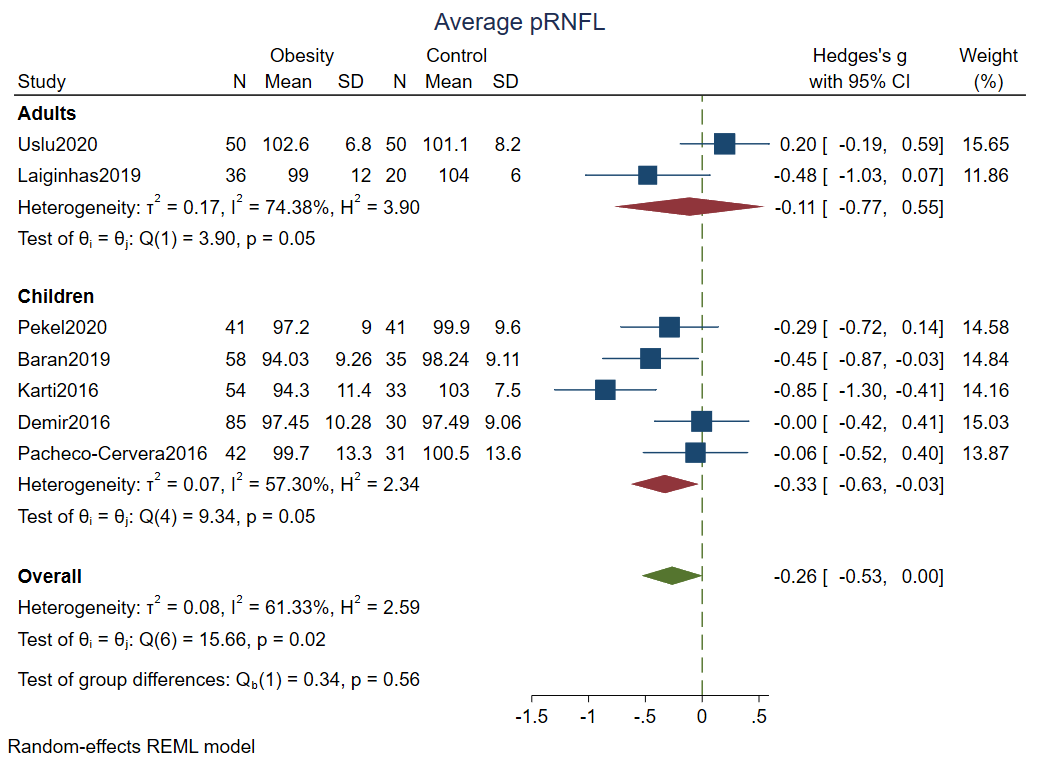
**

**S12 Fig.**

Supplement: S12 Fig — (DOCX) [file pone.0267495.s013.docx]

**
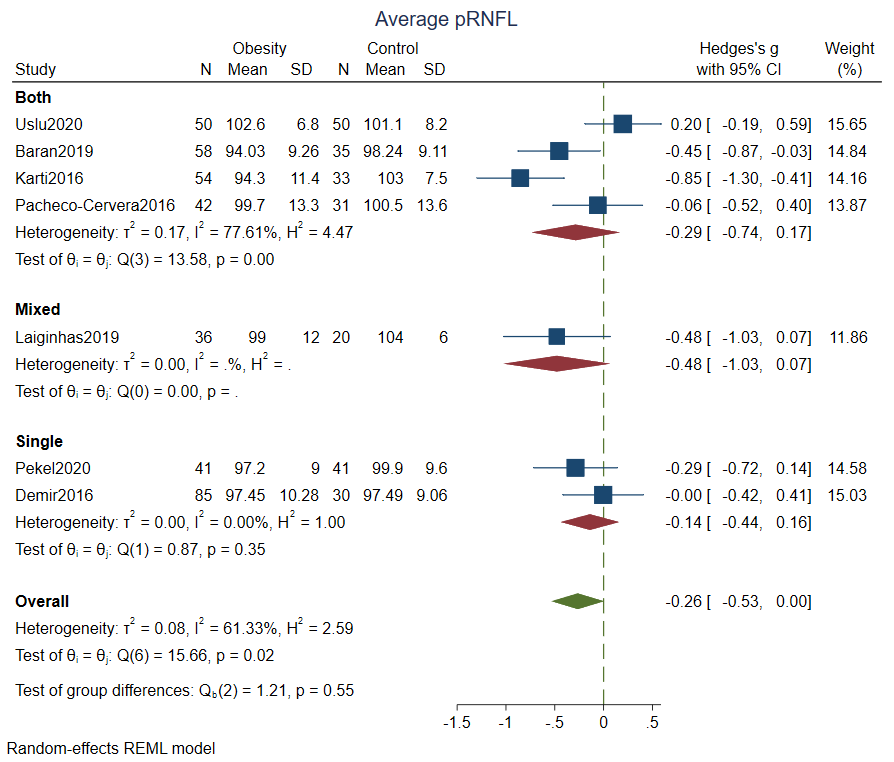
**

**S13 Fig.**

Supplement: S13 Fig — (DOCX) [file pone.0267495.s014.docx]

**
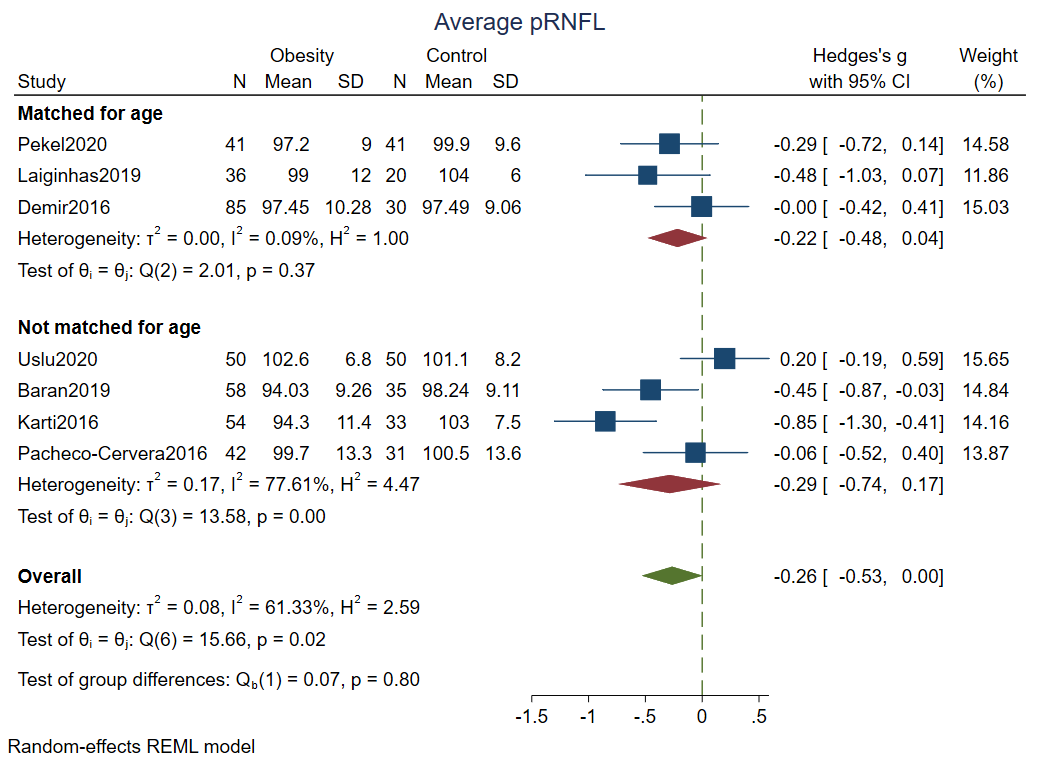
**

**S14 Fig.**

Supplement: S14 Fig — (DOCX) [file pone.0267495.s015.docx]

**
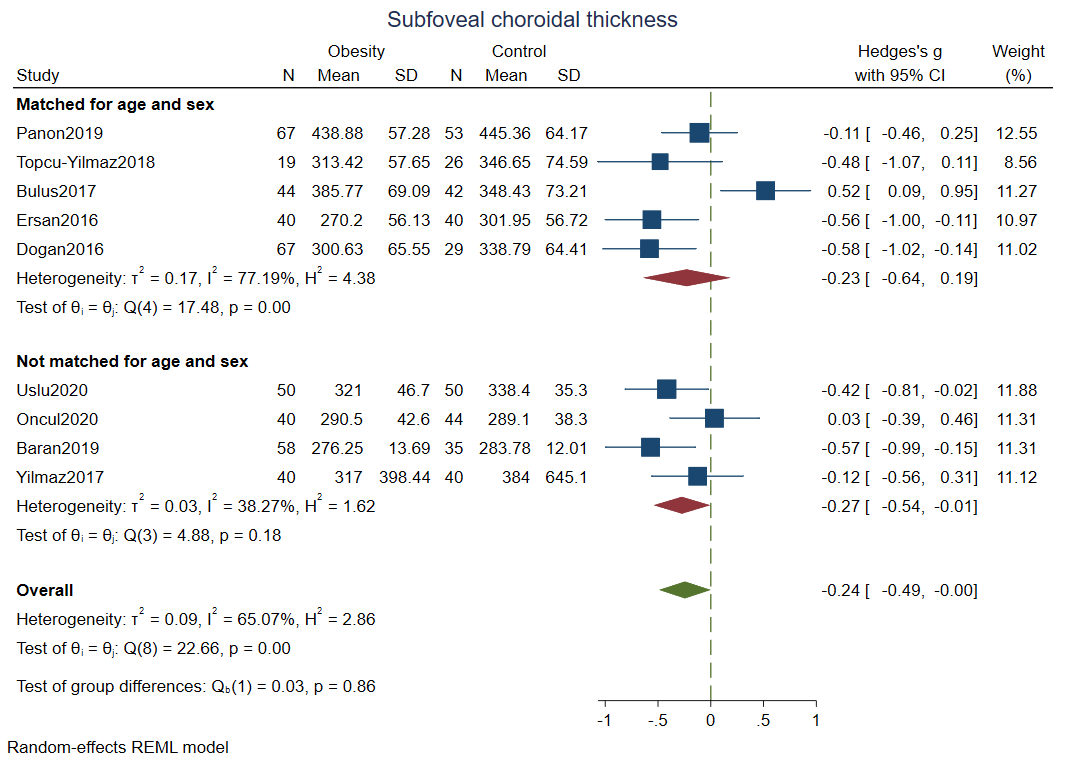
**

**S15 Fig.**

Supplement: S15 Fig — (DOCX) [file pone.0267495.s016.docx]

**
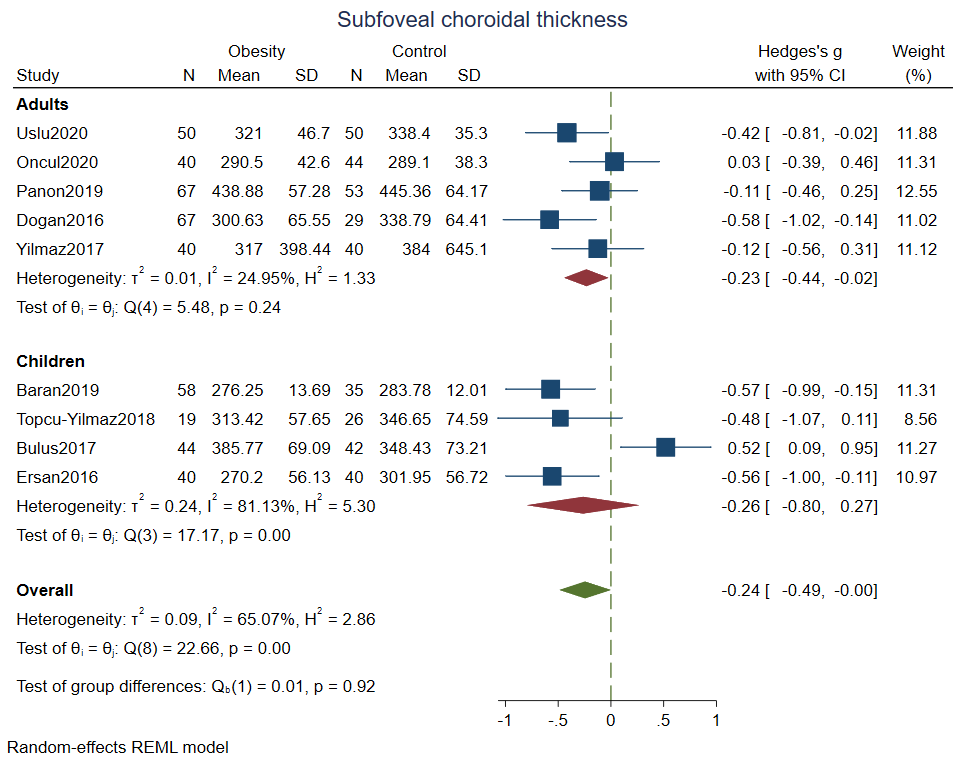
**

**S16 Fig.**

Supplement: S16 Fig — (DOCX) [file pone.0267495.s017.docx]

**
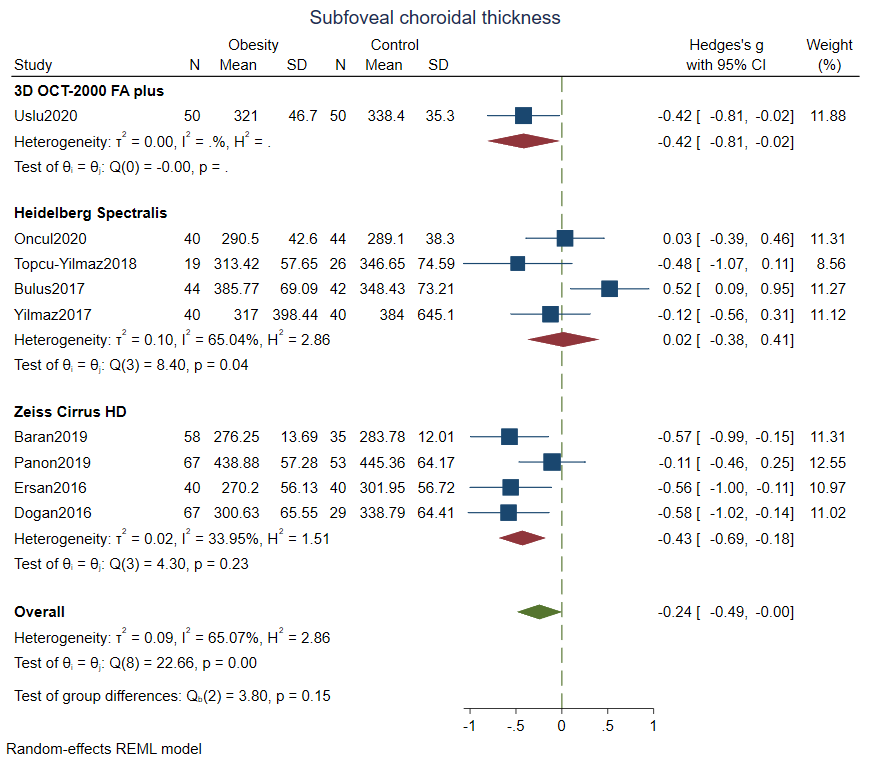
**

**S17 Fig.**

Supplement: S17 Fig — (DOCX) [file pone.0267495.s018.docx]

**
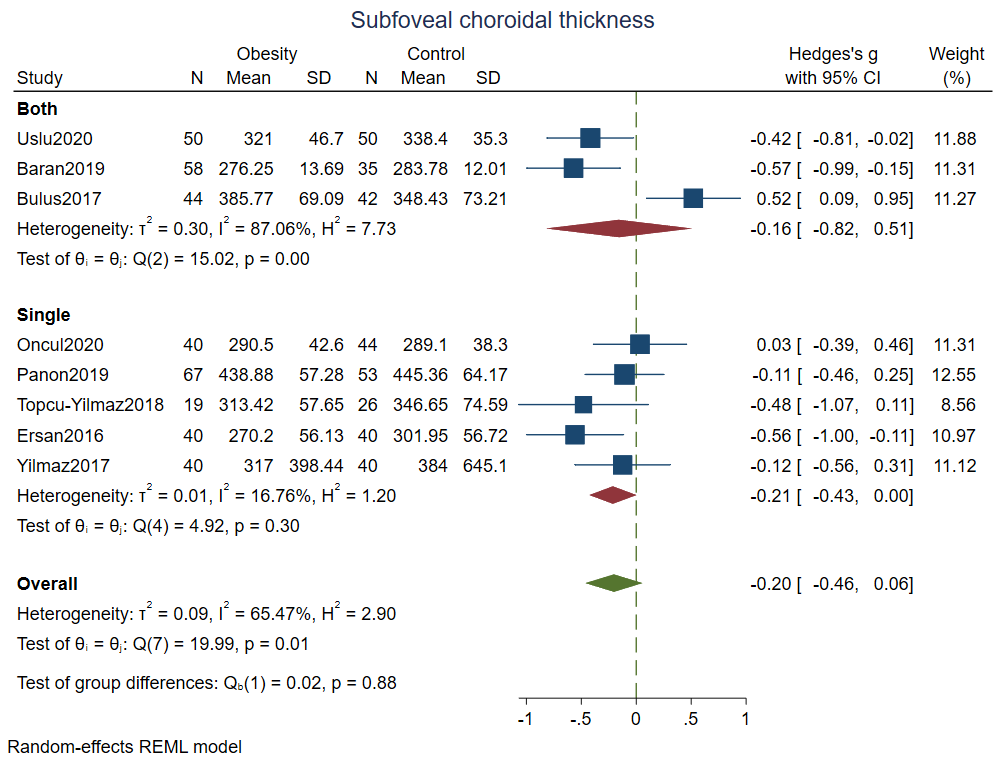
**

**S18 Fig.**

Supplement: S18 Fig — (DOCX) [file pone.0267495.s019.docx]
