# Supplementary material for: Spectral-domain OCT measurements in obesity: A systematic review and meta-analysis
Source: PLoS One. 2022 Apr 27;17(4):e0267495. doi: 10.1371/journal.pone.0267495 (PMC9045631; doi:10.1371/journal.pone.0267495)
Supplement: S1 Table — (DOCX) [file pone.0267495.s020.docx]

**S1 Table.**

| Parameter | Egger’s test  (p-value) | Trim and fill results | | | |
| --- | --- | --- | --- | --- | --- |
|  |  | Initial included studies (n) | Initial SMD [95% CI], p-value | Included + imputed studies (n) | Corrected SMD [95% CI], p-value |
| Macular thickness | 0.01 | 5 | -0.20 [-0.61, 0.22], p=0.36 | 6 | -0.08 [-0.49, 0.33], p=0.72 |
| Average choroidal thickness | 0.01 | 3 | 0.27 [-0.18, 0.72], p=0.24 | 3 | 0.27 [-0.18, 0.72], p=0.24 |

**Abbreviations:** SMD: Standardized mean difference, CI: Confidence interval
