# Supplementary material for: Spectral-domain OCT measurements in obesity: A systematic review and meta-analysis
Source: PLoS One. 2022 Apr 27;17(4):e0267495. doi: 10.1371/journal.pone.0267495 (PMC9045631; doi:10.1371/journal.pone.0267495)
Supplement: S2 Table — (DOCX) [file pone.0267495.s021.docx]

**S2 Table.**

|  | Selection | | | | | Comparability | | | Exposure | | |
| --- | --- | --- | --- | --- | --- | --- | --- | --- | --- | --- | --- |
| Study  First author (year) | Case definition adequacy | Representative of cases | Selection of controls | Definition of controls | Subtotal | Age | Sex | Subtotal | Ascertainment of exposure | Non-response rate | Subtotal |
| Öncül (2021) | * |  | * | * | 3 |  |  | 0 | * | * | 2 |
| Uslu Dogan (2020) | * | * | * | * | 4 | * | * | 2 | * | * | 2 |
| Pekel (2020) |  | * | * | * | 3 | * | * | 2 | * | * | 2 |
| Teberik(2019) |  |  |  | * | 1 | * | * | 2 | * | * | 2 |
| Panon(2019) |  |  | * | * | 2 | * | * | 2 | * | * | 2 |
| Laiginhas(2019) |  | * |  | * | 2 | * |  | 1 | * | * | 2 |
| Baran(2019) |  |  | * | * | 2 | * | * | 2 | * | * | 2 |
| Topcu-Yilmaz(2018) | * |  |  | * | 2 | * | * | 2 | * | * | 2 |
| Özen (2018) | * | * |  | * | 3 |  |  | 0 | * | * | 2 |
| Öner (2018) |  | * |  | * | 2 |  |  | 0 | * | * | 2 |
| Koca(2017) | * |  | * | * | 3 |  |  | 0 | * | * | 2 |
| Karti(2017) |  |  |  | * | 1 |  |  | 0 | * | * | 2 |
| Bulus(2017) | * |  | * | * | 3 | * | * | 2 | * | * | 2 |
| Yumusak(2016) | * |  | * | * | 3 |  |  | 0 | * | * | 2 |
| Ersan(2016) | * |  |  | * | 2 | * | * | 2 | * | * | 2 |
| Dogan(2016) |  |  |  | * | 1 | * | * | 2 | * | * | 2 |
| Demir(2016) |  |  |  | * | 1 | * | * | 2 | * | * | 2 |
| Yilmaz(2015) | * | * | * | * | 4 |  |  | 0 | * | * | 2 |
| Pacheco-Cervera(2015) | * | * | * | * | 4 |  |  | 0 | * | * | 2 |
